# Expression of apoptotic and proliferation factors in gastric mucosa of patients with systemic sclerosis correlates with form of the disease

**DOI:** 10.1038/s41598-019-54988-0

**Published:** 2019-12-05

**Authors:** Katarina Boric, Snjezana Mardesic, Dusanka Martinovic Kaliterna, Mislav Radic, Ivana Tadin Hadjina, Katarina Vukojevic, Ivona Kosovic, Ivana Solic, Sandra Zekic Tomas, Mirna Saraga-Babic

**Affiliations:** 1 Department of Internal Medicine, University Hospital Center Split, Split, Croatia; 2 Department of Anatomy, Histology and Embryology, University of Split, School of Medicine, Split, Croatia; 3 Department of Pathology, University Hospital Center Split, Split, Croatia

**Keywords:** Cell death and immune response, Systemic sclerosis

## Abstract

Despite high prevalence of patients with gastric disease in systemic sclerosis (SSc), its pathogenesis is still poorly understood. We immunohistochemically analysed biopsies of gastric mucosa (GM) in 5 controls and 15 patients with different forms of SSc: limited cutaneous (lc), diffuse cutaneous moderate (sys1) and severe (sys2). The number of positive cells was analysed by a Kruskall-Wallis test, *P* < 0.05 was considered statistically significant. Percentage of proliferating (Ki-67 positive) cells was highest in sys1 (3% in superficial and 4,6% in deeper parts of GM), which dropped to 1% in sys2. Percentage of α-smooth muscle actin (α-SMA) positive cells was 5% in controls, 9% in superficial GM, while in deeper GM rose from 7% to 19% in sys1 and sys2, thus indicating increased myofibroblast population. Caspase-3 positive apoptotic cells characterized 1,5–2% of controls, 8% of superficial and 6% of deeper GM cells in sys1. In sys2, apoptosis affected 50% of surface epithelial and gland cells and 30% of deeper glands, and correlated with increased fibrosis and decreased syndecan-1 expression. Our data demonstrate that sys1 is the most „active” proliferating form of SSc. Sys2 characterize collagen deposition, surface epithelium defects, extensive apoptosis and low proliferation, GM atrophy and loss of function.

## Introduction

Systemic sclerosis (SSc) is a connective tissue disease characterized by the microvascular damage, dysregulation of innate and adaptive immunity, and generalized fibrosis of the skin and internal organs^1^. After skin, gastrointestinal (GI) tract is the most affected organic system involved in approximately 90% of patients with SSc and associated with significant morbidity and reduced quality of life^2^. However, despite numerous studies, etiology and pathogenesis of the disease has not been completely clarified.

Although fibrosis is a hallmark of SSc, sclerotic changes can be classified into the early and late stages. In the early stage, infiltrates of activated T cells produce inflammatory cytokines which cause tissue damage by cytotoxicity, or induce cell apoptosis^3^. A number of investigations, ranging from animal models to human pathology support the view that apoptosis plays an important role in the development of autoimmunity^4^, both in the early and latter pathological changes in SSc. Namely, characteristics of the later stages of SSc is fibroblast resistance to apoptosis^5^, leading to impaired angiogenesis and fibrosis^6,7^. By now, histological investigations on the stomach samples in patients with SSc have proven pronounced deposition of collagen, the presence of myofibroblasts, increased expression of several pro-fibrotic factors^8^, while immunological activation has been demonstrated by the presence of CD4 + lymphocytic infiltrates in gastric mucosa^9^. Several studies dealing with cell proliferation in SSc have shown contradictory results, from higher proliferative potential^10^, to no different potential for SSc cells^11^.

Thus, by now no study has compered apoptosis and proliferation of stomach samples in SSc patients with individuals without SSc. Apoptosis is known to be a form of caspase (cysteine aspartate-specific proteases) mediated cell death, and associated with specific morphological characteristics. Thus, apoptosis can be elucidated by immune-localization with caspase-3 antibody^12^, while Ki-67 protein serves as excellent marker for determining the so-called growth fraction in a given cell population^13^.

In addition, proliferation is regulated by syndecan-1, a member of a large family of transmembrane proteins expressed predominantly in epithelial tissues, but also important in regulation of cell–cell and cell–extracellular matrix interactions, inflammation and wound healing^14^. A good marker of myofibroblasts, found during tissue repair and fibrosis, is α-smooth muscle actin (α-SMA), a cytoskeletal protein primarily localized in vascular smooth muscle cells^15^. As SSc patients have increased collagen and extracellular matrix synthesis driven by transformation of fibroblasts into myofibroblasts, this process can be detected by increased α-smooth muscle actin expression^16^.

In the present study, we investigated involvement of proliferation and apoptosis processes in gastric mucosa of patients with different forms of SSc and individuals without SSc by analyzing expression of proliferation marker Ki-67, apoptotic marker caspase-3, as well as expression of syndecan-1, which is normally involved in maintenance and restoration of epithelial sheet morphology and integrity. We also co-localized α-SMA (myofibroblast marker) with Ki-67 (proliferation marker) to confirm increased proliferation of myofibroblasts in SSc patients.

## Materials and Methods

### Patients

Our study included 15 patients with SSc, diagnosed according to the criteria of the American College of Rheumatology/European League Against Rheumatism^17^ who visited our centre for follow-up care, and 5 volunteers without SSc matched for age and sex, after they had given written informed consent.

The patients were selected after exclusion of patients presenting with other autoimmune disease, malignancy, infection, patients treated with immunosuppressive therapy, intravenous immunoglobulins or use of more than 10 mg/day prednisone or equivalent within the 6 months before inclusion. The disease was classified as diffuse cutaneous SSc (sys1 and sys2) or limited cutaneous SSc (lc) based on the extent of skin involvement according to LeRoy *et al*.^18^. Skin involvement was evaluated using the modified Rodnan skin score (mRSS)^19^, performed by two observers. The severity of organ involvement was evaluated using Medsger’s severity scale for each organ as well as a total Medsger’s severity score summing each scale for a maximum score of 36^20^. All patients underwent a physical examination and comprehensive laboratory evaluation including a full blood count and measurement of the erythrocyte sedimentation rate, renal and liver tests and level of complement. Besides listed, we gathered the following patient characteristics at the time of tissue sampling: gender, age, disease duration measured from the onset of the first symptoms consistent with SSc, presence of digital ulcers, joint/tendon, muscle, renal, vascular, cardiac and gastrointestinal involvement, diffusing capacity for carbon monoxide (DLCO) expressed as percentage of predicted values, left ventricular ejection fraction and estimated pulmonary artery systolic pressure (sPAP) by Doppler echocardiography. From the patients’ medical records data, we collected information on the presence of interstitial lung disease (ILD) on high-resolution CT scan. The presence of SSc-related serum antinuclear antibodies (ANA) was detected by indirect immunofluorescence on Hep-2 cells, while anticentromere antibodies (ACA) and anti-topoisomerase I antibodies (anti Scl 70) were determined by enzyme-linked immunosorbent assay (ELISA).

### Tissue processing

The study was approved by the Ethical Committee of the School of Medicine, University of Split (Class: 003-08/19-03/0003; No.: 2181-198-03-04-19-0009) and Ethical and Drug Committee of the Clinical Hospital Split (Class: 500-03/18-01/55; No.: 2181-147-01/06/M.S.-18-2). All methods were performed in accordance with the relevant guidelines and regulations.

A total of twenty stomach tissue biopsies (15 SSc and 5 controls) were collected by upper gastrointestinal endoscopy. Endoscopic mucosal resection (EMR) was performed on the corpus of stomach by pincers (Olympus Medical Systems Co Ltd, Tokyo, Japan). Tissue pieces were fixed in 4% paraformaldehyde in phosphate buffer and dehydrated in 100% ethanol. They were paraffin embedded and processed as we described previously^21,22^. Haematoxylin and Eosin staining was used on every 10^th^ section to confirm appropriate tissue preservation.

For Mallory trichrome staining, sections were incubated with hematoxylin for 5 minutes, differentiated with tap water, treated with acid fuchsin for 1 minute, rinsed in distilled water and differentiated with 1% phosphomolybdic acid for 1 minute. Sections were stained with Anilin blue for 15 minutes, rinsed with distilled water, differentiated with 1% acetic acid for 1–5 minutes and dehydrated in ethanol and xylol.

Analysis was performed by the fluorescence microscope (Olympus BX61, Tokyo, Japan) equipped with digital camera DP71 (Olympus, Tokyo, Japan). ImageJ Software and Adobe Photoshop were used for further image analyses.

### Immunohistochemistry and immunofluorescence

Tissue sections were deparaffinised and treated as previously described^23^, incubated with primary antibody - rabbit anti-human/mouse active caspase-3 antibody (1:1500; AF835, R&D Systems Inc., Minneapolis, MI, USA), washed in PBS, incubated with a biotinylated secondary antibody (UniTect ABC Kit, Oncogene, Boston, MA, USA) for 30 min, rinsed again in PBS and incubated with avidin biotinylated horseradish peroxidase complex (ABC) for 30 min. Following washing in PBS they were stained with diaminobenzidine (DAB) substrate for 10 min, rinsed again in distilled water, counterstained with Haematoxylin, dehydrated in ethanol and xylene, and cover-slipped.

Cells reacting to caspase-3 primary antibody had brown stained nuclei or their apoptotic fragments. Sections incubated without primary antibodies were used as negative controls.

Two observers with consideration of inter-observer variation analysed the labelling.

Images were captured with digital camera (SPOT Insight, Diagnostic Instruments, USA) mounted on an Olympus BX51 microscope using the SPOT software.

For single immunofluorescence staining, sections were incubated overnight in humidified chamber with primary antibody - mouse monoclonal anti-human syndecan-1 antibody (1:100; B-A38, Abcam, Cambridge, UK).

Combination of primary antibodies for double immunofluorescent antibody staining were rabbit monoclonal anti-human Ki-67 antibody (1:500; ab16667, Abcam, Cambridge, UK) and mouse monoclonal anti-human α-SMA antibody (1:100; M0851, DAKO, Glostrup, Denmark).

Sections were washed in PBS and incubated with combination of secondary antibodies: donkey anti-mouse IgG H&L Alexa Fluor (1:500, ab150105, Abcam, Cambridge, UK), donkey anti-rabbit IgG H&L Alexa Fluor 488 (1:500, ab150073, Abcam, Cambridge, UK) and goat anti-mouse IgG H&L TRITC (1:400, ab6786, Abcam, Cambridge, UK) for one hou, rinsed in PBS, counterstained with DAPI for 2 minutes, washed again in PBS and cover-slipped (Immuno-mount, Shandon Inc, Pittsburgh, PA, USA).

### Statistics

The number of cells was evaluated quantitatively by two independent investigators and classified as negative (no stained) or positive (stained) cells.

For Ki-67 and α-SMA analyses photomicrographs were obtained using a fluorescence microscope and DP-SOFT Version 3.1 software. Tissue sections were divided in areas 50 µm × 50 µm at × 40 magnification. The α-SMA and Ki-67 positive cells were counted in at least 25 squares. The percentage of caspase-3 immunoreactive cells was calculated from total number of cells in superficial glands and surface epithelium, and in deep glands. Only the strong brown staining intensity was considered positive to active caspase-3. The percentage of positive cells was calculated and expressed as mean ± SD. Data were tested with Kruskall-Wallis test followed by Dunn’s post-hoc test (GraphPad Software, La Jolla, CA, US). Statistical significance level was set at p < 0.05.

## Results

### Patients demographics

Fifteen patients with SSc were enrolled in this study (Table 1). All patients were female and Caucasion. The patients mean age was 54 ± 13 years and their mean disease duration from the onset of the first symptoms consistent with SSc was 13 years (range 1–35). With respect to the disease subset, ten were classified as diffuse cutaneous SSc (dcSSc) and five limited cutaneous SSc (lcSSc). According to the Medsger’s severity score five patients were classified as mild, five moderate diffuse cutaneous (sys1) and five severe diffuse cutaneous disease (sys2).

**Table 1:** Clinical and serological characterstics of SSc patients.

Characteristic		Value
Age (years)^a^		54.3 ± 13.60
Gender	Female	15 (100)
SSc duration (years)^b^		13 (1–35)
SSc subset	lcSSc	5 (33.33)
	dcSSc	10 (66.66)
Autoantibody pattern	ANA positivity	13 (86.66)
	ACA	4 (26.66)
	Anti-Scl 70	9 (60.00)
mRSS^b^		15 (4–26)
GI tract involvement	Present	10 (66.66)
Joint/tendon involvement	Present	12 (80.00)
Muscular involvement	Present	10 (66.66)
Digital ulcers	Present	3 (20.00)
Peripheral vascular involvement	Present	12 (80.00)
Interstitial lung disease	Present	8 (53.33)
DLCO^a^		58.44 ± 22.16
Heart involvement	Present	3 (20.00)

^a^Values expressed as mean ± standard deviation. ^b^ Values expressed as median with minimum-maximum range. Values expressed as absolute number (%) unless otherwise indicated. SSc, systemic sclerosis; lcSSc, limited cutaneous systemic sclerosis; dcSSc, diffuse cutaneous systemic sclerosis; ANA, antinuclear antibodies; ACA, anticentromere antibodies; Anti-Scl 70, anti-topoisomerase I antibodies; mRSS, modified Rodnan skin score; GI, gastrointestinal; DLCO, diffusing capacity for carbon monoxide (% predicted).

### Haematoxylin and eosin and caspase-3 staining (1a)

#### Control samples

**Figure 1:**
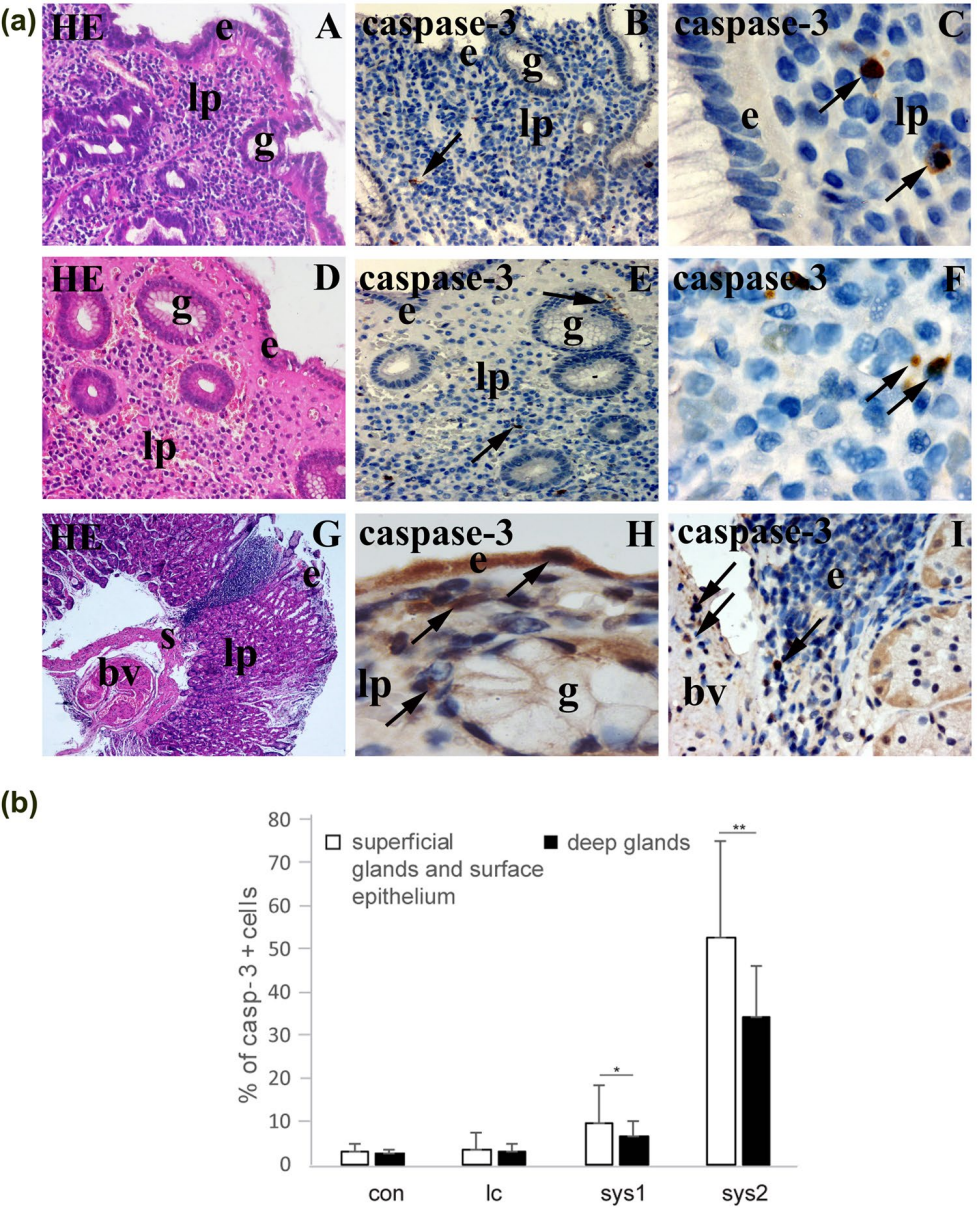
(**a**) Haematoxylin and eosin staining and staining to caspase-3. Gastric mucosa consists of: covering cylindrical epithelium (e), connective tissue of lamina propria (lp), profiles of gastric glands (g) and apoptotic cells (arrows) (A). In control samples, several apoptotic cells with brown staining cytoplasm are observed in both surface epithelium, glands and lamina propria (B,C). Gastric mucosa of patients with limited cutaneous systemic sclerosis additionally shows vacuolated cells and hyalinisation in lamina propria, as well as increased number of apoptotic cells within the glands and connective tissue (D–F) Gastric mucosa of patients with diffuse cutaneous systemic sclerosis shows numerous inflammatory cells, abnormal blood vessels (bv) in submucosa (s) and huge number of apoptotic cells in the surface epithelium, glands, lamina propria and wall of blood vessels (G–I). Haematoxylin and eosin staining (A,D,G), immunohistochemical staining to caspase-3 (B,C,E,F, H,I). (**b**) *Percentage of caspase-3 positive cells in superficial glands and surface epithelium, and deeper glands of gastric mucosa in control samples and different forms of systemic sclerosis*. Data are expressed as the mean ± standard deviation (vertical line). Significant differences are indicated by *p < 0.01, **p < 0.001, ***p < 0.0001, Kruskall-Wallis.

Normal gastric mucosa of control samples stained with haematoxylin and eosin (H&E) consists of cylindrical epithelium and connective tissue of lamina propria, which contains superficial gastric pits and glands covered by pale staining mucous cells (Fig. 1a), and deeper parts of glands covered by different cells types, including chief cells and parietal cells (not shown).

When stained with caspase-3, gastric mucosa occasionally shows brown staining nuclei of apoptotic cells primarily in the lamina propria and rarely in the surface epithelium of glands (Fig. 1b,c).

#### Samples of patients with limited cutaneous systemic sclerosis (lc)

Gastric mucosa of patients with lc (H&E staining) shows homogenous hyalinised appearance of the lamina propria between the surface epithelium and profiles of superficial glands, with reduction of cells in the affected area (Fig. 1d).

Staining with caspase-3 reveals several brown-stained nuclei of apoptotic cells both in the lamina propria and superficial glands and at the margins of the hyalinised zone (Fig. 1e).

Higher magnification of lamina propria shows numerous sparkling (vacuolated), apoptotic cells and bodies (Fig. 1f).

#### Samples of patients with moderate (sys1) and severe (sys2) diffuse cutaneous systemic sclerosis

Gastric mucosa and submucosa of patients with sys1 and particularly sys2 forms of SSc shows defects of surface epithelium and accumulation of inflammatory cells (lymphocytes) that extends throughout the whole depth of mucosa. Submucosa contains abnormally changed blood vessels (Fig. 1g). Staining with caspase-3 discloses apoptotic cells in surface epithelium, superficial and deep glands and lamina propria, (Fig. 1h). Numerous apoptotic cells are observed in the area of lymphocyte accumulation and in the wall of big vessels in the submucosa as well (Fig. 1i).

### Percentage of caspase-3 positive cells (1b)

Percentage of apoptotic (caspase-3 positive) cells was analysed in the epithelial cells of covering epithelium, superficial and deep glands and lamina propria of gastric mucosa in control, lc, sys1 and sys2 samples.

Control samples (con) of both superficial and deeper parts of lamina propria, covering and gland epithelium contained low number of apoptotic cells (1,5–2%).

In *limited cutaneous systemic sclerosis(lc)*, number of apoptotic cells rose to only 3% in superficial parts of gastric mucosa, and to 2% in its deeper parts. In *diffuse sys1 form* gastric mucosa number of apoptotic calls increased to 8% in its superficial parts, and to 6% in its deeper parts. In *diffuse sys2 form*, surface parts of gastric mucosa contained 50% of apoptotic cells. Such high number of apoptotic cells were associated by big defects of covering epithelium, which seem to be the result of extensive apoptosis of cylindrical lining epithelium. Deeper parts of gastric mucosa contained 30% of apoptotic cells, which were primarily localized in deep glands (affecting both chief and parietal cells).

### Mallory staining

#### Control samples

**Figure 2:**
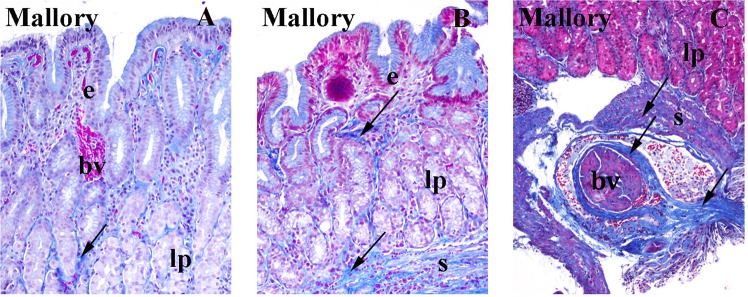
*Mallory stainin*g for detection for detection of collagen (arrows) in the gastric mucosa of control (**A**), limited cutaneous (**B**) and diffuse(**C**) cutaneous forms of systemic sclerosis: epithelium (e), lamina propria (lp), submucosa (s), blood vessel (bv). Note increasingly blue staining of collagen (arrows) in lamina propria and submucosa from control towards diffuse systemic sclerosis.

Mallory staining of the same control samples shows light-to- moderate blue staining of connective tissues collagen in the lamina propria. Mucous content of the surface epithelium and glands is also stained light blue, while the erythrocytes are stained red (Fig. 2a).

#### Samples of patients with limited cutaneous systemic sclerosis (lc)

Mallory staining shows moderate blue staining of accumulated collagen in the lamina propria and strong collagen staining in the submucosa (Fig. 2b).

#### Samples of patients with moderate (sys) and severe (sys2) diffuse cutaneous systemic sclerosis

Mallory staining shows very strong staining of collagen in the lamina propria and submucosa, which contains blood vessels with abnormally thickened intima (Fig. 2c).

### Immunofluorescence staining to syndecan-1

#### Control samples

**Figure 3:**
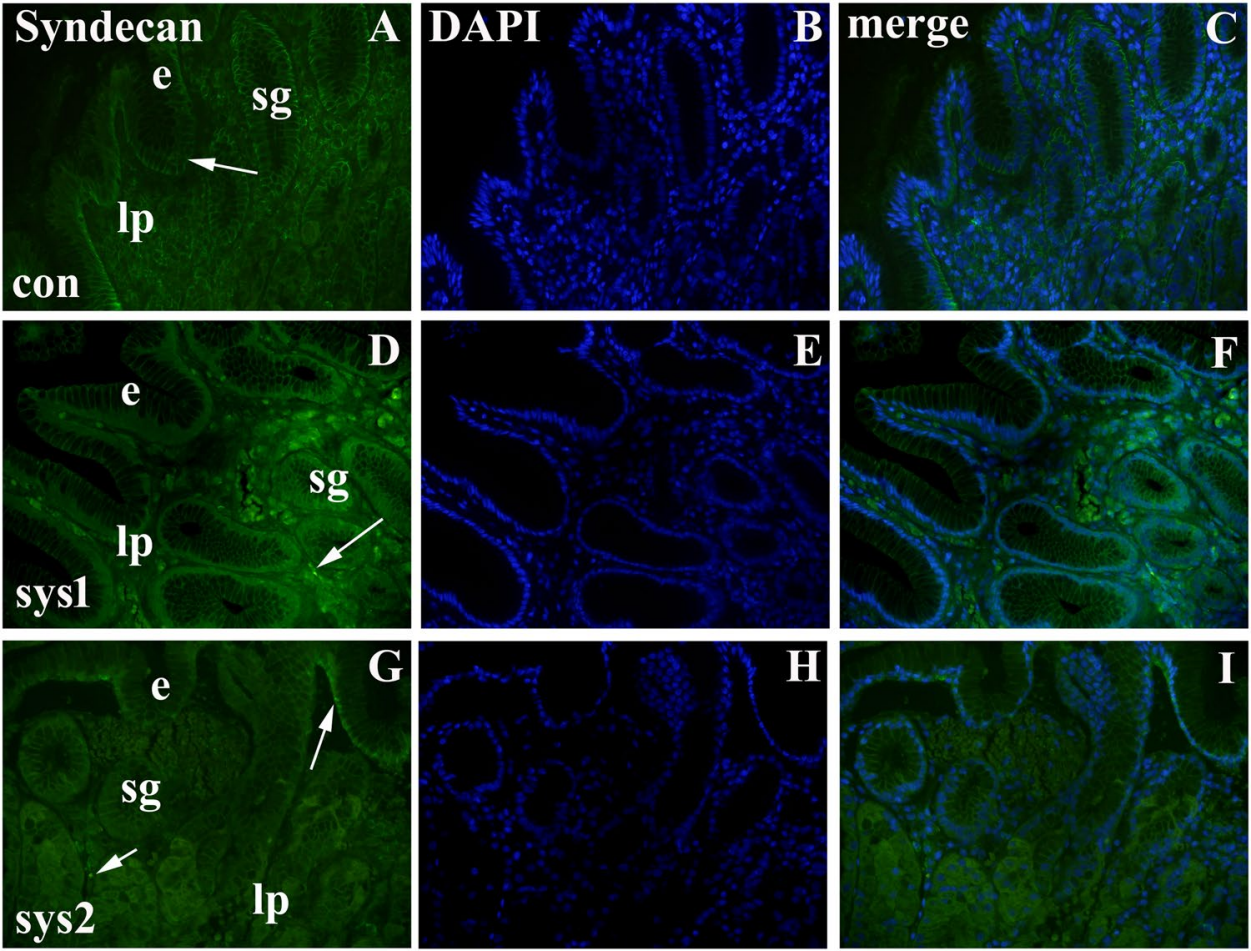
Immunfluorescence staining to syndecan-1. Gastric mucosa of control samples (con) (**A**–**C**), moderate diffuse cutaneous (sys1) and severe diffuse cutaneous (sys2) forms of systemic sclerosis: epithelium (e), lamina propria (lp), superficial gastric glands (sg), reactivity to syndecan-1 (arrows). Note decrease in syndecan-1 expression in samples of diffuse cutaneous systemic sclerosis compared to control samples.

The gastric mucosa of the *control samples (con)* shows immunoreactivity to syndecan-1 in surface gastric epithelium, superficial glands and lamina propria, while deep glands of gastric mucosa are negative to syndecan-1 (Fig. 3a–c).

*Limited cutaneous systemic sclerosis(lc)* shows staining pattern similar to control gastric mucosa (not shown).

#### Moderate diffuse cutaneous systemic sclerosis (sys1)

In the gastric mucosa of patients with sys1, syndecan-1 immunoreactivity is absent in the surface epithelium except weakly in the basal parts of some cells. Syndecan-1 immunoreactivity is only occasionally observed in basal parts of surface glands and in some cells of lamina propria (Fig. 3d–f).

#### Severe diffuse cutaneous systemic sclerosis (sys2)

In the gastric mucosa of patients with severe form of SSc, immunoreactivity to syndecan-1 is present only very weakly in basal part of some surface epithelial cells and occasionally in cells of lamina propria. Syndecan-1 expression is absent in superficial and deep glands of gastric mucosa (Fig. 3g–i).

### Double immunofluorescence staining to Ki-67 and α-SMA (4a)

#### Control samples

**Figure 4:**
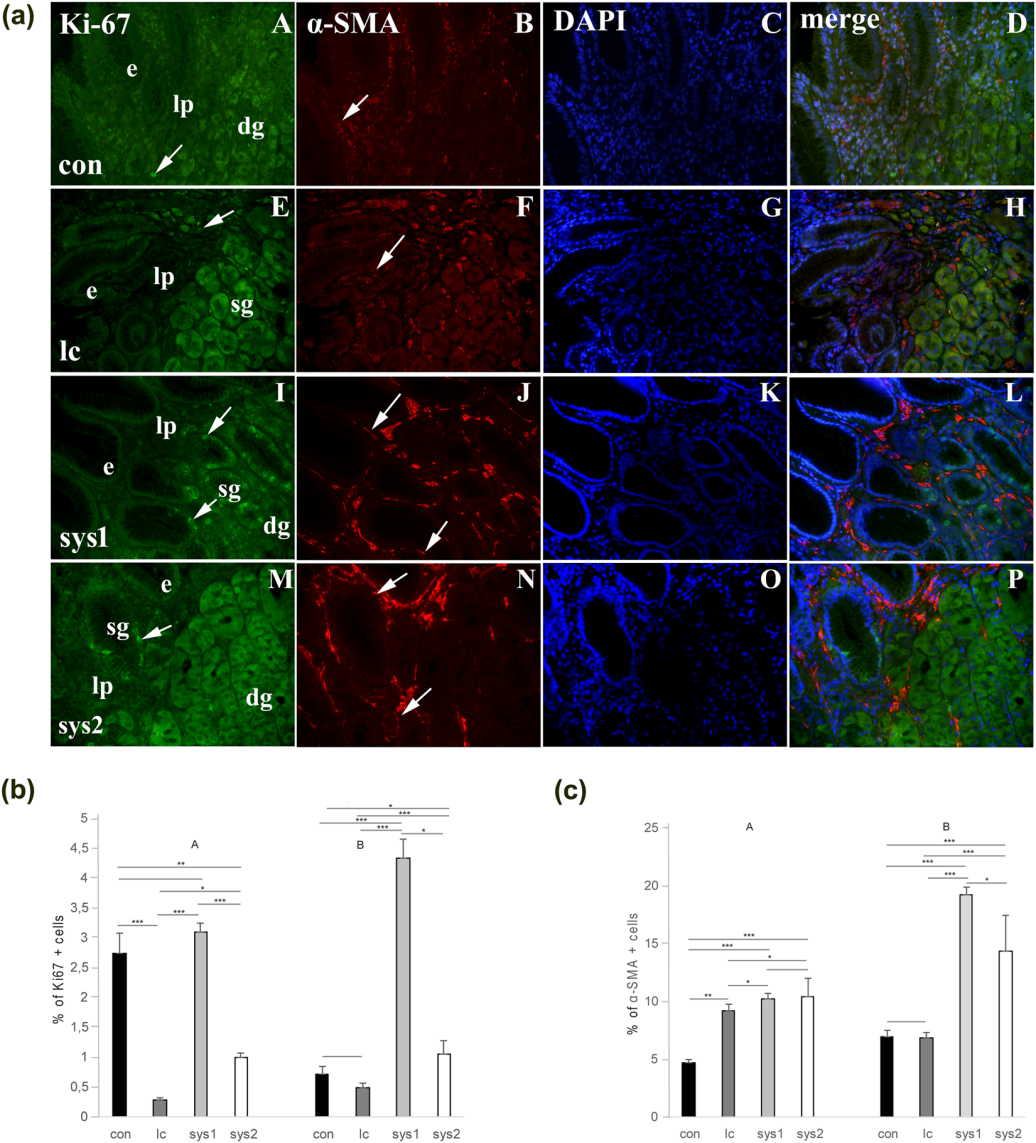
Double immunofluorescence staining to Ki-67 and α-SMA. (**a**) Distribution and co-expression of proliferation marker (Ki-67) and maker for myofibroblasts and myoblasts (α-SMA) in gastric mucosa of control samples (con) (A–D), limited cutaneous systemic sclerosis (lc) (E–H), moderate diffuse cutaneous systemic sclerosis (sys1) (I–L) and severe (sys2) forms of diffuse cutaneous systemic sclerosis (M–P): epithelium (e), lamina propria (lp), superficial glands (sg), deep glands (dg), Ki-67 or α-SMA positive cells (arrows). Note increase in expression of both markers in patients with systemic sclerosis, particularly in moderate diffuse cutaneous form (sys1). (**b**) *Percentage of Ki-67 positive cells in the superficial glands and surface epithelium (A) and deeper glands (B) of gastric mucosa in control samples and different forms of systemic sclerosis* (**c**). *Percentage of α-SMA positive cells in superficial parts of lamina propria (A) and deeper parts of lamina propria of gastric mucosa in control samples and different forms of systemic sclerosis*. Legends: control (con); limited cutaneous (lc); moderate diffuse cutaneous (sys1); severe diffuse cutaneous (sys2) systemic sclerosis. Significant differences are indicated by *p < 0.01, **p < 0.001, ***p < 0.0001 (Kruskall-Wallis test).

In the gastric mucosa of *control samples (con)*, Ki-67 proliferating cells are only occasionally seen in the lamina propria and superficial glands (Fig. 4a), while strong α-SMA staining is observed in the lamina propria, corresponding to myofibroblasts or myoblasts of blood vessels (Fig. 4b). DAPI nuclear stain characterizes all nuclei in the gastric mucosa, except proliferating cells (Fig. 4c). Merging of the three staining reveals that Ki-67 nuclear stain is not co-expressed in the same cells that express α-SMA (Fig. 4d), meaning that neither fibroblasts nor myoblasts in the walls of blood vessels proliferate in control gastric mucosa.

#### Limited cutaneous systemic sclerosis (lc)

Slightly increased number of proliferating Ki-67 positive cells is observed in the lamina propria and superficial glands of gastric mucosa (Fig. 4e). α-SMA staining characterizes both increased number of fibroblasts and myoblasts in the walls of blood vessels localized in the lamina propria (Fig. 4f). DAPI nuclear stain characterizes all cell nuclei except those stained with Ki-67 (Fig. 4g). Merging of the three staining discloses that majority of α-SMA positive cells do not proliferate (do not express Ki-67), except for a small population of cells in the lamina propria and vascular wall (Fig. 4h).

#### Moderate diffuse cutaneous systemic sclerosis (sys1)

Numerous proliferating Ki-67 positive cells are observed in the lamina propria, superficial and deep gastric glands (Fig. 4i). Abundant profiles of α-SMA positive cells are seen in the lamina propria (Fig. 4j), while blue DAPI staining characterizes nuclei of all cells except Ki-67 positive (Fig. 4k). Merging of different staining shows that Ki-67 mostly does not co-expressed in the population of cells positive to α-SMA (Fig. 4l).

#### Severe diffuse cutaneous systemic sclerosis (sys2)

In this sample, Ki-67 positive cells are less numerous than in sys1 sample. They are predominantly localized in superficial glands, but also in deep glands and lamina propria (Fig. 4m). α-SMA cells are seen in lamina propria (Fig. 4n), while DAPI stains the cell nuclei, except Ki-67 positive ones (Fig. 4o). Merging of different staining reveals absence of co-expression of Ki-67 and α-SMA, indicating absence of proliferation of myofibroblasts and myoblasts in the walls of blood vessels (Fig. 4p).

### Percentage of Ki-67 positive cells (4b)

Proliferation of the cells *in the surface parts of lamina propria and surface glands* (A) of control group (healthy mucosa or mucosa of patients without SSc) was 2,7%, while in the lc it was only occasionally seen (less than 1%). In the sys1, proliferation increased in both lamina propria and surface glands to 3%, while in sys2 form dropped to 1%.

Proliferation in the *deeper parts of lamina propria and deep gastric glands* (B) was 0,6% for the control group, while it was 0,5% in the lc. In sys1, proliferation increased to 4,6% in both connective tissue of lamina propria and deep glands, while in sys2 proliferation dropped to 1%.

### Percentage of, α-SMA positive cells (4c)

In the *surface area of lamina propria* (A) of control group the percentage of α-SMA positive cells was between 4 and 5%, while in lc form of systemic sclerosis there were 9% of α-SMA positive cells. In diffuse cutaneous sclerosis, both in sys1 and sys2 forms, percentage of α-SMA positive cells was 10%.

In *deeper area of lamina propria* (B) of control samples, percentage of α-SMA positive cells was 7%, while the same percentage characterized lc as well. In diffuse cutaneous form of systemic sclerosis, deeper parts of lamina propria contained 19% of α-SMA positive cells in sys1, while in sys2 form it was 14%.

## Discussion

Despite high prevalence and considerable morbidity in patients with gastric SSc, its pathogenesis is still poorly understood. Hypothesis for the pathogenesis of gastrointestinal involvement in SSc usually includes vasculopathy, autoimmunity and autonomic neuropathic disturbance which can occur as a primary event, or secondarily to vasculopathy and fibrosis^24^. Our study showed that with advancement of SSc, imbalance in ratio of proliferation and apoptosis in the epithelial and connective tissue cells gradually led to loss of surface and deep glands, defects of surface epithelial lining, increased accumulation of connective tissue collagen, vascular changes as well as decreased expression of syndecan-1 in the whole gastric mucosa. Previous studies revealed generalized fibrosis affecting all gastric wall layers, pronounced deposition of collagen, and presence of myofibroblasts and markers of immune activation and immuno-inflammation^8,9,25^. In addition, scleroderma colonic fibrosis mouse model confirmed that many of the structural or motility abnormalities may be the result of smooth muscle atrophy, fibroblast dysfunction associated with over-reactivity of TGF beta and related fibrogenic pathways^26^. Taroni and al (2015) demonstrated that deregulated molecular programs responsible for SSc are similar in different end organs^27^. In our study, unlike controls, SSc gastric samples displayed either mild or severe inflammation within both surface and deep parts of gastric mucosa and submucosa. Those findings were accompanied by the increased cell proliferation and in some cases presence of enlarged-vessels (megacapillaries), characterised by thickening of the walls in submucosal vessels. In addition, numerous proliferating cells characterized gastric glands and connective tissue in limited and in diffuse moderate form of SSc, while in severe forms of SSc proliferation dropped, probably due to increased cell apoptosis. This could indicate that increased proliferation might be a compensatory process primarily affecting gland cells, but also connective tissue fibroblasts which later on underwent differentiation into myofibroblasts. Tsujino and al (2017) also showed increased myofibroblast proliferation in the vessel walls of lungs in mouse model of SSc^28^. We demonstrated that changes in the gastric mucosa of SSc features proliferative (active) and non-proliferative phase, as described in skin and by videocapillaroscopy^29^.

Gastric mucosa of SSc patients compared to healthy individuals showed increased number of apoptotic cells in epithelium, surface glands and lamina propria, which in diffuse forms of scleroderma characterized also deep glands and walls of blood vessels. Described epithelial apoptosis probably led to surface defects, absence of protective layer of mucus and problems with absorption. In addition, mucosal microcirculation with delivery of oxygen and nutrients, and removal of toxic substances is also essential for maintaining mucosal integrity^30^.

Co-existence of GI and pulmonary fibrosis in patients with SSc suggests that GI involvement could contribute to the natural history of pulmonary fibrosis^31^. The impairment of deep glands by extensive apoptosis could lead to hypoacidity and improper enzyme production. In addition, significant increase in content of connective tissue is associated with gastric dysmotility, significant weight loss and functional morbidity^32^.

Besides detecting differential distribution of apoptotic and proliferating cells in gastric mucosa, our study also showed reduced expression of syndecan-1 in SSc patients compared to healthy individuals. Syndecan-1 seems to be involved not only in regulation of proliferation and apoptosis processes, but also in migration, angiogenesis and inflammatory cell recruitment^33^. However, despite its importance in maintenance and restoration of epithelial sheet integrity, the involvement of syndecan-1 in pathogenesis of SSc received only modest attention^34^. Our study showed differential expression patterns of syndecan-1 in different forms of SSc, thus confirming gradual loss of its role in control of proliferation and apoptotic processes with advancement of disease.

In our study we also found apoptotic cells throughout all layers of submucosal blood vessels. In previous studies, apoptosis of endothelial cells was widely explored in skin and serum of SSc animal models and patients. Besides in the skin, Sgonc and al (1996) demonstrated presence of endothelial cell apoptosis in oesophagus, lungs and kidneys. Endothelial cell apoptosis preceded perivascular inflammatory infiltrates and collagen deposition in SSc^35^.

Thus, proliferation and apoptosis patterns presented in this study resemble those reported for the skin of SSc patients, with reduction of proliferation and significantly higher caspase-3 protein levels in SSc dermal microvascular endothelial cells^36^. This implies similar pathogenesis of SSc processes in different organs.

In our study, we have been able to demonstrate that moderate diffuse SSc expresses “active” form of disease course associated with numerous proliferating cells, while severe diffuse SSc is marked by collagen deposition, partial loss of epithelial layer, extensive apoptosis of surface and deep glands, and low proliferation. Therefore, apoptosis and proliferation accompanied by inflammation seems to be important histopathological characteristic of gastric mucosa in SSc, leading to atrophy and loss of function.
